# Unique molecular signatures as a hallmark of patients with metastatic breast cancer: Implications for current treatment paradigms

**DOI:** 10.18632/oncotarget.1946

**Published:** 2014-05-02

**Authors:** Jennifer J. Wheler, Barbara A. Parker, Jack J. Lee, Johnique T. Atkins, Filip Janku, Apostolia M. Tsimberidou, Ralph Zinner, Vivek Subbiah, Siqing Fu, Richard Schwab, Stacy Moulder, Vicente Valero, Maria Schwaederle, Roman Yelensky, Vincent A. Miller, M Philip J. Stephens, Funda Meric-Bernstam, Razelle Kurzrock

**Affiliations:** ^1^ Department of Investigational Cancer Therapeutics (Phase I Program), The University of Texas MD Anderson Cancer Center, Houston, TX; ^2^ Center for Personalized Cancer Therapy and Division of Hematology and Oncology, University of California at San Diego Moores Cancer Center; ^3^ Department of Biostatistics, The University of Texas MD Anderson Cancer Center, Houston, TX; ^4^ Department of Breast Medical Oncology, The University of Texas MD Anderson Cancer Center, Houston, TX; ^5^ Center for Personalized Cancer Therapy, Moores Cancer Center, University of California San Diego, La Jolla, CA; ^6^ Foundation Medicine, Cambridge MA

**Keywords:** Genomics, Breast Cancer, PI3K, Clinical Trials

## Abstract

Our analysis of the tumors of 57 women with metastatic breast cancer with next generation sequencing (NGS) demonstrates that each patient's tumor is unique in its molecular fingerprint. We observed 216 somatic aberrations in 70 different genes, including 131 distinct aberrations. The most common gene alterations (in order of decreasing frequency) included: *TP53*, *PIK3CA*, *CCND1*, *MYC*, *HER2 (ERBB2)*, *MCL1*, *PTEN*, *FGFR1*, *GATA3*, *NF1*, *PIK3R1*, *BRCA2*, *EGFR*, *IRS2*, *CDH1*, *CDKN2A*, *FGF19*, *FGF3* and *FGF4*. Aberrations included mutations (46%), amplifications (45%), deletions (5%), splices (2%), truncations (1%), fusions (0.5%) and rearrangements (0.5%), with multiple distinct variants within the same gene. Many of these aberrations represent druggable targets, either through direct pathway inhibition or through an associated pathway (via ‘crosstalk’). The ‘molecular individuality’ of these tumors suggests that a customized strategy, using an “N-of-One” model of precision medicine, may represent an optimal approach for the treatment of patients with advanced tumors.

Genomic profiling demonstrates a multitude of aberrations across a broad spectrum of cancer-related genes [1, 2] and represents a new way to classify cancer [3]. Many studies demonstrate that matching patients to treatment based on molecular characteristics can result in remarkable responses, even in advanced, refractory disease, despite heterogeneity in patients' molecular landscapes [3]. However, most patients have short-lived responses.

Herein, we demonstrate that, amongst 57 patients with metastatic breast cancer, no two patients had the same molecular portfolio. The number of distinct aberrations driving advanced tumors may therefore be responsible for the remarkable resistance of metastatic malignancies to prolonged remission or cure, especially with our current therapeutic strategies [3]. These data suggest that traditional design paradigms governing diagnosis and management of metastatic cancer and the clinical research that informs them, all of which are predicated on grouping patients in order to evaluate targeted agents, may be inadequate to address the complexity unveiled by modern genomics.

We used next generation sequencing (NGS) of 182 to 236 genes to interrogate tumors from 57 women with metastatic breast cancer (26 with hormone receptor (HR)-positive and HER2-negative tumors; 7 with HR-positive/HER2-positive tumors; 4 with HR-negative/HER2-positive tumors; and, 20 with triple negative breast cancer (TNBC) (Supplemental Table 1) [4]. Importantly, if different variants in each gene were considered as distinct then each patient had a unique profile of genomic aberrations (Supplemental Table 1). Even if variants within a gene were considered identical, there were 54 different genomic landscape patterns amongst the 57 patients (Supplemental Table 1).

The most commonly altered genes (in order of decreasing frequency) were: *TP53*, *PIK3CA*, *CCND1*, *MYC*, *HER2 (ERBB2)*, *MCL1*, *PTEN*, *FGFR1*, *GATA3*, *NF1*, *PIK3R1*, *BRCA2*, *EGFR*, *IRS2*, *CDH1*, *CDKN2A*, *FGF19*, *FGF3* and *FGF4* (Supplemental Table 2). The most frequent aberrations were mutations and amplifications, comprising 46% and 45% of abnormalities, respectively. The average number of molecular aberrations per patient was 4 (average = 4 in HR-positive/HER2-negative; 4 in HR-positive/HER2-positive; 3 in HR-negative /HER2-positive; and 4 in TNBC (Figure 1).

**Figure 1:**
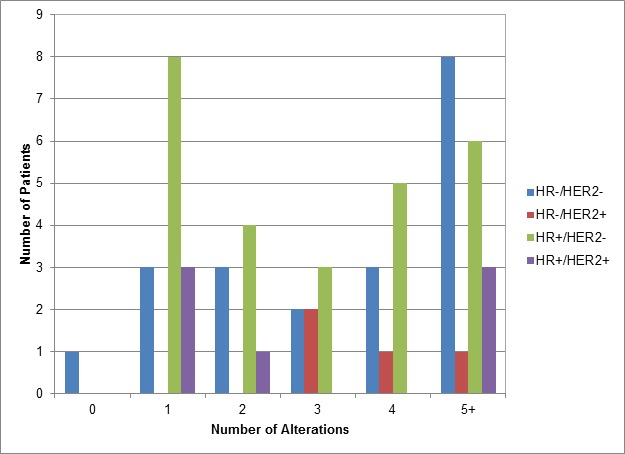
Number of Alterations seen in Patients Grouped by Hormone Receptor (HR) and HER2 Status

A total of 131 distinct somatic aberrations (216 aberrations in total) were identified: 80 mutations (including mutation variants in the same gene); 37 amplifications; five deletions; five splices; two truncations; one fusion; and, one rearrangement. Of the 80 distinct mutation variants we observed (Supplemental Table 3), 49 have not been previously reported for patients with breast cancer in COSMIC, The Cancer Genome Atlas (TCGA) [1, 2] and other large genomic databases [5, 6]. Thirty-seven distinct amplifications were found, the most common being *CCND1* (12 patients), *MYC* (12 patients), *MCL1* (7 patients), *HER2* (*ERBB2*; 7 patients), and *FGFR1* (6 patients). All amplification had been previously reported in breast cancer.

Many, if not most, of the genomic aberrations represent actionable targets, either by approved drugs or by agents in clinical trials [7, 9-14]. Among the most common aberrations were those seen in the PI3K/AKT/mTOR pathway (Supplemental Table 2) [7, 8]: *PIK3CA* mutations with eight different variants were noted (15 patients, including 3 patients with more than one variant); *PIK3CA* amplifications (3 patients); *PTEN* deletions (6 patients); *PIK3R1* mutation (5 patients with 4 different mutation variants); *AKT3* amplifications (2 patients); and *PTEN* or *AKT1* mutation (1 patient each). Other druggable aberrations include, but are not limited to, EGFR, HER2, CDKN2A, FGFR/FGF family, KRAS, MDM2, BRCA2, and more.

Our observations are consistent with the hypothesis that metastatic tumors, even when originating in the same organ (e.g., the breast), are mostly distinct at the molecular level (malignant snowflakes) [9]. Of special interest, many of the genomic aberrations we observed may be actionable, through either direct or indirect ‘cross talk’ signal suppression [7, 9-14]. For instance, prior literature indicates that *PIK3CA* is the oncogene showing the highest frequency of gain-of-function mutations in breast cancer [7, 10-12] (ranging from ~13% in TNBC to ~42% in HR positive+/HER2- subgroup) [10]. In our study, *PIK3CA* was the second most common aberrant gene, and was involved in 12% of abnormalities (Supplemental Table 2). Further, if patients with other molecular alterations in the PI3K/AKT/mTOR pathway are included (e.g. *PTEN*, *AKT*, *PIK3R1* etc), the subset of patients expands. Of therapeutic interest, the BOLERO-2 study of the hormone modulator exemestane and the mTOR inhibitor everolimus showed that patients whose tumors had 0 or 1 genomic aberrations in *PIK3CA* or *FGFR* pathways or in *CCND1* had better outcomes [13]. Further, we previously reported that heavily pretreated patients with *PIK3CA*-mutant breast and gynecologic tumors have higher than expected response rates (partial/complete response rates ~30%) when mTOR inhibitor-based combination regimens are administered [11, 14, 15]. However, despite the large percentage of aberrations (Supplemental Table 2) that can be targeted by drugs that are already approved or in clinical trials, our data illustrates why current clinical trials and practice generally provide only short-lived tumor regressions. In particular, the fact that most patients have multiple aberrations, and that the abnormalities differ from individual to individual, suggests that treating different patients with the same drug or drug combination may be insufficient to optimize success.

There are limitations to our data: the number of patients we analyzed was small and, in some cases, different aberrations in the same gene may have a similar effect on the downstream pathway. However, even if we considered all aberrations in the same gene as functionally equivalent (a highly unlikely situation), 95% (54 of 57) of our patients still had distinct genomic portfolios. The reality that NGS interrogation of these tumors has revealed is that, since most patients with metastatic breast malignancies have unique genomic landscapes, the standard methods deployed for therapeutic prosecution of molecular aberrations may need to be revised.

Classic clinical trial design mandates grouping patients by tumor site and histology (e.g., breast or colon cancer) and evaluating the efficacy of a drug or combination of agents. In the era of molecular therapeutics, the first generation of newer clinical trial designs stratify patients, often on the basis of a single molecular aberration as identified by a companion diagnostic, and test the efficacy of a cognate inhibitor to improve outcomes. The latter model represents a scientifically rational advancement that has been successful to an extent. Indeed, patients with HER2-positive breast cancer respond to trastuzumab, lapatinib, and pertuzumab [16], and *ALK*-rearranged advanced lung tumors respond to the *ALK* inhibitor crizotinib [17]. However, the majority of patients do not achieve durable responses. It is not surprising that these tactics fail to provide long-term survival benefit since our current observations suggest that each patient, despite the commonality of the anomaly that stratifies them for the clinical trial/treatment, has a distinct aberrant molecular backdrop. Indeed, these novel clinical trials using companion diagnostics to select patients so that an appropriately targeted agent can be tested are still retrofitting our new knowledge about metastatic cancer–at the molecular level, each patient has a unique and complex tumor–into a dated clinical trial paradigm where patients are grouped together to evaluate a drug.

Previously, we have shown that, using genomic aberrations to match patients with advanced, heavily-pretreated malignancies (including breast cancer) to drugs, in the context of phase I studies, resulted in improved outcomes as assessed by response rate, progression-free survival and overall survival, as well as progression-free survival on matched Phase I therapy as compared to last conventional therapy; in the case of melanoma, progression-free survival on matched phase I therapy was improved as compared to first-line treatment [8, 11, 18, 19]. Of interest, these studies used the first-generation molecular profiling technology available at the time (i.e. tumors were probed for gene aberrations one at a time, rather than with next generation sequencing). Further trials employing next generation sequencing, as well as additional advanced omic technologies, and randomized designs are warranted.

In summary, NGS may represent a disruptive technology that mandates a new approach to clinical research and cancer treatment. Even if there is some convergence of pathways with functional impact [20-23], introduction of additional omics tests–transcriptomics, proteomics, epigenomics etc.–may reveal yet more complexity and differences between patients' tumors leading to major challenges in personalized treatment. The complexity of the molecular genetics of metastatic cancer is not conducive to the success of classic drug-centric clinical trial models, where patients are grouped together in order to test the efficacy of a drug or combination. We propose testing a new patient-centric, molecular matching strategy to find an optimal treatment regimen tailored to each patient's genomic profile acquired from multi-assay molecular testing. Importantly, this approach would permit the therapy given to vary from individual to individual consistent with N-of-One customization. The framework for such a trial would utilize genomics and/or other omic technologies to navigate patients to individualized therapy. Forerunners of this design have been utilized in the IMPACT/PREDICT trials (NCT00851032) [8], as well as newer trials such as WINther (the latter utilizing both genomics and transcriptomics to type patients) (NCT01856296) and the planned NCI MATCH trial [24]. These trials concentrated on finding matches for single aberrations; next generation trials can exploit the same design, but deploy advanced multiplex technology, and focus on appropriate tailored combinations.

## METHODS

Fifty-seven sequential consented patients with advanced, pathologically confirmed breast cancer who had next generation sequencing (182 to 236 genes) were analyzed. Tumor samples were evaluated for genomic alterations including base substitutions, short insertions and deletions, amplifications, homozygous deletions, gene fusions, truncations and rearrangements (Foundation Medicine, Cambridge, MA). DNA was extracted from 40 *μ*m of FFPE tissue (minimum 20% tumor cells) using the Maxwell 16 FFPE Plus LEV DNA Purification kit (Promega) and quantified using a standardized PicoGreen fluorescence assay (Invitrogen). Library Construction was performed using 50-200 ng of DNA sheared by sonication to ~100-400 bp before end-repair, dA addition and ligation of indexed, Illumina sequencing adaptors. Enrichment of target sequences (all coding exons of 182 or 236 cancer-related genes and selected introns from 14 or 19 genes recurrently rearranged in cancer) was achieved by solution-based hybrid capture with custom biotinylated oligonucleotide baits. Enriched libraries were sequenced to an average median depth of >500X with 99% of bases covered >100X (Illumina HiSeq 2000 platform using 49 × 49 paired-end reads) and mapped to the reference human genome (hg19) using the Burrows-Wheeler Aligner and the publicly available SAMtools, Picard and Genome Analysis Toolkit. Point mutations were identified by a Bayesian algorithm; short insertions and deletions, determined by local assembly; gene copy number alterations (amplifications), by comparison to process matched normal controls; and gene fusions/rearrangements, by clustering chimeric reads mapped to targeted introns. Local site permissions to use clinical samples were also obtained.

## SUPPLEMENTARY TABLES


