# Supplementary material for: Unique molecular signatures as a hallmark of patients with metastatic breast cancer: Implications for current treatment paradigms
Source: Oncotarget. 2014 May 2;5(9):2349–54. doi: 10.18632/oncotarget.1946 (PMC4058010; doi:10.18632/oncotarget.1946)
Supplement: Supplementary file 1 [file oncotarget-05-2349-s001.pdf]

Unique molecular signatures as a hallmark of patients with metastatic breast cancer: Implications for current treatment paradigms

SUPPLEMENTARY MATERIALS

Supplemental Table 1: Patient Characteristics and Molecular Profiling of 57 Breast Tumors

Case No.	Age	ER/PR/ HER2	No of prior therapies	Histology	Molecular Results	Examples of Possible Cognate Targeted Therapies*
1	41	+/-/-	5	Ductal	EGFR amplification	EGFR targeting with erlotinib [25]or lapatinib [26]
2	54	+/+/+	7	Ductal	ERRB2 amplification<br>ERBB3 amplification<br>NF1 splice 6705 A>G<br>NF1 truncation<br>PIK3CA mutation H1047R<br>CCND1 amplification<br>MDM2 amplification<br>MYC amplification<br>MYCL1 amplification<br>TP53 mutation R248Q	ERBB2 targeting with trastuzumab or lapatinib [27]<br><br>NF1 targeting with mTOR inhibitor everolimus [28] and/or MEK inhibitor trametanib [29]<br><br>PIK3CA targeting with mTOR inhibitor everloimus [11, 30]<br><br>CCND1 targeting with CDK4/6 inhibitor palbociclib [31]
3	51	+/+/-	15	Ductal	ERRB2 fusion ERBB2-GRB7<br>PIK3CA mutation H1047R<br>CCND1 amplification<br>CDH1 mutation Q23*	ERBB2 targeting with trastuzumab or lapatinib [27]<br><br>PIK3CA targeting with mTOR inhibitor everolimus [11, 30]<br><br>CCND1 targeting with CDK4/6 inhibitor palbociclib [31]
4	68	+/+/-	7	Lobular	PIK3CA mutation H1047R<br>CDH1 mutation T823fs*23	PIK3CA targeting with mTOR inhibitor everolimus [11, 30]
5	43	-/- /+	8	Ductal	ERBB2 amplification<br>PIK3CA mutation N345K<br>TP53 mutation G245S	ERBB2 targeting with trastuzumab or lapatinib [27]<br>PIK3CA targeting with mTOR inhibitor everolimus [11, 30]

Case No.	Age	ER/PR/ HER2	No of prior therapies	Histology	Molecular Results	Examples of Possible Cognate Targeted Therapies*
6	45	+/+/-	6	Ductal	CCND1 amplification<br>FGFR1 amplification<br>ARID1A mutation Q708*	CCND1 targeting with CDK4/6 inhibitor palbociclib [31]<br><br>FGFR1 targeting with pazopanib, lenvatinib or lucitanib [32]
7	49	+/+/-	7	Ductal	CCND1 amplification	CCND1 targeting with CDK4/6 inhibitor palbociclib [31]
8	52	+/-/-	14	Ductal	CCND1 amplification<br>ESR1 mutation L536H	CCND1 targeting with CDK4/6 inhibitor palbociclib [31]<br><br>ESR1 targeting with the estrogen receptor downregulator fulvestrant [33]
9	41	-/-/-	5	Ductal	TP53 splice site 559+1 G>A	Not clear though Said et al [34] showed longer PFS with bevacizumab in patients with p53 mutations
10	37	+/+/-	7	Ductal	PIK3CA mutation H1047L	PIK3CA targeting with mTOR inhibitor everolimus [11, 30]
11	55	+/+/-	3	Ductal	PIK3CA mutation H1047R<br>EPHB1 mutation A922T	PIK3CA targeting with mTOR inhibitor everolimus [11, 30]
12	54	-/-/-	5	Metaplastic	PIK3R1 mutation N453_T454insNN<br>PTEN mutation K327fs*16<br>MCL1 amplification<br>TP53 mutation N131del<br>KDM6A mutation Q692fs*37<br>RB1 splice site 1421+1G>C	PIK3CA and PTEN targeting with mTOR inhibitor everolimus [11, 30]<br><br>MCL1 targeting with sorafenib [35]
13	52	+/+/-	9	Lobular	CDH1 mutation Q511fs*10	Not clear
14	45	+/+/-	5	Ductal	NF1 mutation 1679fs*21<br>IRS2 amplification<br>TP53 mutation R248Q	NF1 targeting with mTOR inhibitor everolimus [28] and/or MEK inhibitor trametanib [29]<br><br>Not clear though Said et al [34] showed longer PFS with bevacizumab in patients with p53

Case No.	Age	ER/PR/ HER2	No of prior therapies	Histology	Molecular Results	Examples of Possible Cognate Targeted Therapies*
						mutations
15	59	-/-/-	6	Ductal	FGFR1 amplification<br>FGFR2 amplification<br>CCND1 amplification<br>MAP2K2 amplification<br>MYC amplification,<br>TP53 mutation C242fs*5	FGFR targeting with pazopanib, lenvatinib or lucitanib [32]<br><br>CCND1 targeting with CDK4/6 nhibitor palbociclib [31]
16	64	-/-/-	11	Ductal	NO ALTERATIONS	Not clear
17	50	-/-/-	2	Metaplastic	NF2 mutation K159fs*16<br>TP53 mutation F109fs*39	NF2 targeting with mTOR inhibitor everolimus [36]<br><br>Not clear though Said et al [34] showed longer PFS with bevacizumab in patients with p53 mutations
18	61	-/-/-	5	Unknown	TP53 mutation R273H<br>CDK8 amplification<br>MYC amplification<br>ATM mutation K1698R<br>SOX10 mutation S431L	Not clear though Said et al [34] showed longer PFS with bevacizumab in patients with p53 mutations
19	58	+/+/-	5	Ductal/ Lobular	PIK3CA mutation H1047R	PIK3CA targeting with mTOR inhibitor everolimus [11, 30]
20	43	+/-/-	5	Ductal	CCNE1 amplification<br>TP53 mutation I195T<br>IRS2 amplification<br>MCL1 amplification	MCL1 targeting with sorafenib [35]
21	37	+/+/-	8	Ductal	CCND1 amplification<br>FGFR1 amplification<br>PRKDC rearrangement	CCND1 targeting with CDK4/6 inhibitor palbociclib [31]<br><br>FGFR1 targeting with pazopanib, lenvatinib or lucitanib [32]
22	45	+/+/-	9	Ductal	PIK3CA mutation H1047R<br>PIK3R1 mutation G376R<br>CCND1 amplification<br>FGFR1 amplification<br>MYC amplification<br>MCL1 amplification<br>FBXW7 mutation D112E	PIK3CA targeting with mTOR inhibitor everolimus [11, 30]<br><br>CCND1 targeting with CDK4/6 inhibitor palbociclib [31]

Case No.	Age	ER/PR/ HER2	No of prior therapies	Histology	Molecular Results	Examples of Possible Cognate Targeted Therapies*
						FGFR targeting with pazopanib, lenvatinib or lucitanib [32]<br><br>MCL1 targeting with sorafenib [35]
23	56	+/+/-	8	Ductal	PIK3CA mutation H1047R<br>CCND1 amplification<br>MCL1 amplification<br>EMSY amplification<br>FGF19 amplification<br>FGF3 amplification<br>FGF4 amplification	PIK3CA targeting with mTOR inhibitor everolimus [11, 30]<br><br>CCND1 targeting with CDK4/6 inhibitor palbociclib [31]<br><br>MCL1 targeting with sorafenib [35]<br><br>FGFR targeting with pazopanib, lenvatinib or lucitanib [32]
24	45	-/-/-	4	Metaplastic	PIK3CA amplification<br>PTEN deletion<br>FGFR2 amplification<br>MYC amplification<br>TP53 mutation R273C	PIK3CA (PTEN) targeting with mTOR inhibitor everolimus [11, 30]<br><br>FGFR targeting with pazopanib, lenvatinib or lucitanib [32]
25	33	-/-/-	5	Ductal	PIK3CA amplification<br>RET amplification<br>AURKA amplification<br>MCL1 amplification<br>MYC amplification<br>SOX2 amplification<br>IRS2 amplification<br>TP53 splice site 673-2 A>G	PIK3CA targeting with mTOR inhibitor everolimus [11, 30]<br><br>RET targeting with sorafenib or cabozantib [37, 38]<br><br>MCL1 targeting with sorafenib [35]
26	38	-/-/-	6	Ductal	PTEN deletion<br>RPTOR amplification<br>BRCA1 mutation Q1447*<br>CCND3 amplification<br>SMAD4 deletion<br>TP53 mutation K132R	PTEN loss leads to activation of PIK3CA pathway, which can be targeted with mTOR inhibitor everolimus [30]<br><br>BRCA1 targeting with PARP inhibitor

Case No.	Age	ER/PR/ HER2	No of prior therapies	Histology	Molecular Results	Examples of Possible Cognate Targeted Therapies*
						olaparib [39]
27	56	-/-/-	4	Ductal	PIK3R1 mutation V172fs*7<br>TP53 mutation H179R	PIK3R1 aberration actvates PIK3CA pathway; PIK3CA pathway targeting with mTOR inhibitor everolimus [11, 30]
28	48	+/+/-	3	Metaplastic	PIK3CA mutation H1047R<br>TP53 mutation K320*	PIK3CA targeting with mTOR inhibitor everolimus [11, 30]
29	68	+/-/-	4	Squamous	PIK3CA mutation H1047R<br>PTEN deletion<br>JAK2 amplification<br>MYC amplification<br>TP53 mutation F134S<br>NOTCH1 truncation	PIK3CA and PTEN targeting with mTor inhibitor everolimus [11, 30]<br><br>JAK2 targeting with JAK2 inhibitor ruxolitinib [40]
30	43	-/-/-	4	Metaplastic	PTEN deletion<br>EGFR mutation P848L	PTEN loss leads to activation of PIK3CA pathway, which can be targeted with mTOR inhibitor everolimus [30]<br><br>EGFR targeting with EGFR inhibitor erlotinib [25] or lapatinib [26]
31	52	+/+	10	Ductal	ERBB2 amplification	ERBB2 targeting with trastuzumab or lapatinib [27]
32	33	+/-/-	11	Ductal	PIK3CA mutation E545K<br>KRAS mutation G12S<br>MEN1 mutation I85fs*33<br>GATA3 mutation S438fs*9+	PIK3CA targeting with mTOR inhibitor everolimus [11, 30]<br><br>KRAS targeting with MEK inhibitor trametinib [41]
33	44	=/-/-	6	Ductal	BRCA2 mutation A938fs*21	BRCA2 targeting with PARP inhibitor olaparib [39]
34	61	-/-/-	8	Ductal	PIK3R1 mutation G376R<br>STK11 deletion<br>TP53 mutation M133K<br>TP53 mutation V10I<br>PALB2 mutation S766*<br>MYST3 amplification	PIK3R1 activates PIK3CA pathway; PIK3CA targeting with mTOR inhibitor everolimus [11, 30]<br><br>PALB2 targeting with

Case No.	Age	ER/PR/ HER2	No of prior therapies	Histology	Molecular Results	Examples of Possible Cognate Targeted Therapies*
						mitomycin C [42]
35	68	+/-/-	7	Ductal	ERBB2 amplification<br>PIK3CA mutation Q546R<br>PIK3CA mutation H1047L<br>PIK3CA mutation G1049R<br>RB1 splice site 719-1G>A<br>SMAD4 mutation E330K<br>TP53 mutation F270V	ERBB2 targeting with trastuzumab or lapatinib [27]<br><br>PIK3CA targeting with mTOR inhibitor everolimus [11, 30]
36	53	+/+/+	4	Ductal	AKT3 amplification<br>IKBKE amplification<br>PIK3CA mutation E545K<br>PIK3CA mutation E726K<br>MCL1 amplification<br>MDM4 amplification<br>DAXX mutation S97fs*46<br>HGF amplification	AKT3 and PIK3CA targeting with mTOR inhibitor everolimus [11, 30]<br><br>MCL1 targeting with sorafenib [35]<br><br>HGF is ligand for MET kinase; MET targeting with MET inhibitor crizotinib or cabozantinib [43]
37	47	-/-/-	8	Ductal	FGF14 amplification<br>GATA3 mutation A333fs*20<br>IRS2 amplification	FGFR targeting with pazopanib, lenvatinib or lucitanib [32]
38	56	+/-/-	1	Ductal	BRCA2 mutation N43fs*1<br>BRCA2 mutation V1283fs*2<br>MCL1 amplification,<br>NTRK3 mutation E412K	BRCA2 targeting with PARP inhibitor olaparib [39]<br><br>MCL1 targeting with sorafenib
39	61	-/-/-	5	Ductal	TP53 mutation L114fs*10	Not clear though Said et al [34] showed longer PFS with bevacizumab in patients with p53 mutations
40	23	-/-/-	8	Ductal	ERBB2 amplification<br>ERBB2 mutation V777L<br>TP53 mutation S303fs*42	ERBB2 targeting with trastuzumab<br>or lapatinib [27]
41	69	-/-/-	3	Metaplastic	PIK3R1 mutation Y580fs*19<br>CCND2 amplification<br>CDKN2A deletion<br>FGF23 amplification	PIK3CA pathway targeting with mTOR inhibitor everolimus [11, 30]<br><br>CDKN2A loss leads to activation of the CDK4/6 pathway which can be targeted

Case No.	Age	ER/PR/ HER2	No of prior therapies	Histology	Molecular Results	Examples of Possible Cognate Targeted Therapies*
						with CDK4/6 inhibitor palbociclib [31]<br><br>FGFR targeting with pazopanib, lenvatinib or lucitanib [32]
42	66	-/-/-	1	Metaplastic	NF1 mutation R304*<br>PIK3CA mutation E545K<br>PIK3CA mutation H1047R<br>PTEN deletion<br>EGFR amplification<br>CDKN2A deletion<br>HRAS mutation G12S<br>MYC amplification	NF1 targeting with mTOR inhibitor everolimus [28] and/or MEK inhibitor trametanib [29]<br>PIK3CA and PTEN targeting with mTOR inhibitor everolimus [11, 30]<br><br>EGFR targeting with erlotinib [25] or lapatinib [26]
43	41	+/-/-	4	Ductal	PIK3CA mutation E545K,<br>BRCA2 mutation K3326*<br>CCND1 amplification<br>HRAS mutation G12D<br>GATA3 mutation D336fs*17<br>FGF19 amplification<br>FGF3 amplification<br>FGF4 amplification<br>MYST3 amplification	PIK3CA and PTEN targeting with mTOR inhibitor everoliumus [11, 30]<br><br>CCND1 targeting with CDK4/6 inhibitor palbociclib [31]<br><br>FGFR targeting with pazopanib, lenvatinib or lucitanib [32]
44	44	-/-/-	3	Ductal	CDKN2A mutation Y44fs*1<br>TP53 mutation I195fs*52<br>CCND1 amplification<br>CCND2 amplification<br>FGF19 amplification<br>FGF23 amplification<br>FGF3 amplification<br>FGF4 amplification<br>FGF6 amplification	CCND1 targeting with CDK4/6 inhibitor palbociclib [31]<br><br>FGFR targeting with pazopanib, lenvatinib or lucitanib [32]
45	40	-/-/-	5	Ductal	PIK3CA amplification<br>SOX2 amplification<br>TP53 mutation G302fs*42<br>FLT3 mutation L260*	FLT3 targeting with FLT3 inhibitor sorafenib [44]<br><br>PIK3CA targeting with mTOR inhibitor everolimus [11, 30]
46	48	-/-/-	3	Ductal	AKT1 mutation E17K	AKT1 targeting with mTOR inhibitor

Case No.	Age	ER/PR/ HER2	No of prior therapies	Histology	Molecular Results	Examples of Possible Cognate Targeted Therapies*
						everolimus [45]
47	46	+/+/-	4	Ductal	EGFR amplification<br>CCND1 amplification<br>CDKN2A/B deletion<br>FGFR1 amplification<br>MYC amplification<br>TP53 mutation P151A	EGFR targeting with erlotinib [25] or lapatinib [26]<br><br>CCND1 targeting with CDK4/6 inhibitor palbociclib [31]<br><br>FGFR targeting with pazopanib, lenvatinib or lucitanib [32]
48	51	-/-/+	9	Ductal	ERBB2 amplification<br>PIK3CA mutation H1047L<br>AURKA amplification<br>TP53 mutation R342P<br>CREBBP mutation P858S<br>ZNF217 amplification	ERBB2 targeting with trastuzumab<br>or lapatinib [27]<br><br>PIK3CA targeting with mTOR inhibitor everolimus [11, 30]
49	43	-/-/+	10	Ductal	ERBB2 amplification<br>MYC amplification<br>CDK6 amplification<br>TP53 mutation R213*	ERBB2 targeting with trastuzumab<br>or lapatinib [27]
50	51	+/+/-	4	Ductal	ESR1 mutation Y537S	ESR1 targeting with fulvestrant, which downregulates estrogen receptor fulvestrant [33]
51	54	+/-/+	3	Ductal	GATA3 mutation *445fs*2+	Not clear
52	55	+/+/+	6	Ductal	RET mutation C634R<br>GATA3 mutation P436fs*11+	RET targeting with sorafenib or cabozantinib [37, 38]
53	49	-/-/-	2	Ductal	AKT3 amplification<br>MYC amplification<br>MYCL1 amplification<br>TP53 mutation R248Q	AKT3 targeting with mTOR inhibitor everolimus [45]
54	45	+/?/-	13	Ductal	NF1 mutation R1276Q	NF1 targeting with mTOR inhibitor everolimus [28] and/or MEK inhibitor trametanib [29]
55	35	-/-/-	2	Ductal	PTEN deletion<br>MYC amplification<br>TP53 mutation C238W<br>TP53 mutation S241fs*5	PTEN loss leads to activation of PIK3CA pathway, which can be targeted with mTOR inhibitor everolimus [11, 30]
56	54	+/+/+	4	Ductal	TP53 mutation Y163*	Not clear though Said et al [34] showed

Case No.	Age	ER/PR/ HER2	No of prior therapies	Histology	Molecular Results	Examples of Possible Cognate Targeted Therapies*
						longer PFS with bevacizumab in patients with p53 mutations
57	68	+/+/+	3	Ductal	ERBB2 mutation V777L<br>ERBB2 mutation S1050*<br>FGFR1 amplification<br>PIK3CA mutation E545K<br>TET2 mutation S714*<br>TP53 mutation W53*<br>ZNF703 amplification	ERBB2 targeting with trastuzumab or lapatinib [27]<br><br>FGFR1 targeting with pazopanib, lenvatinib or lucitanib [32]<br><br>PIK3CA targeting with mTOR inhibitor everolimus [11, 30]

*** Examples of possible therapies provided. In many cases, multiple drugs are available that target the pathway; only some examples are shown. Preference was given to drugs as follows: (i) approved for breast cancer, e.g. everolimus, fulvestrant, trastuzumab, and lapatinib; (ii) approved for some type of malignancy, e.g., cabozantinib, crizotinib, erlotinib, pazopanib, ruloxitinib, sorafenib, trametinib, bevacizumab, mitomycin C; (iii) not approved but in advanced studies with clinical evidence of efficacy, e.g., lenvatinib, lucitanib, olaparib, palbociclib**

Supplemental Table 2: Gene List and Frequency of Aberrations (using each aberration as a unit)

Gene	Frequency	Percent (%)
**TP53**	30	13.9
**PIK3CA**	25	11.6
**CCNDA**	12	5.6
**MYC**	12	5.6
**HER2(ERBB2)**	11	5.1
**MCL1**	7	3.2
**PTEN**	7	3.2
**FGFR1**	6	2.8
**GATA3**	5	2.3
**NF1**	5	2.3
**PIK3R1**	5	2.3
**BRCA2**	4	1.9
**EGFR**	4	1.9
**IRS2**	4	1.9
**CDH1**	3	1.4
**CDKN2A**	3	1.4
**FGF19**	3	1.4
**FGF3**	3	1.4
**FGF4**	3	1.4
**AKT3**	2	0.9
**AURKA**	2	0.9
**CCND2**	2	0.9
**ESR1**	2	0.9
**FGF23**	2	0.9
**FGFR2**	2	0.9
**HRAS**	2	0.9
**MYCL1**	2	0.9
**MYST3**	2	0.9
**RB1**	2	0.9
**RET**	2	0.9
**SMAD4**	2	0.9
**SOX2**	2	0.9
**AKT1**	1	0.5
**ARID1A**	1	0.5
**ATM**	1	0.5
**BRCA1**	1	0.5
**CCND3**	1	0.5
**CCNE1**	1	0.5
**CDK6**	1	0.5

**CDK8**	1	0.5
**CDKN2A/B**	1	0.5
**CREBBP**	1	0.5
**DAXX**	1	0.5
**EMSY**	1	0.5
**EPHB1**	1	0.5
**ERBB3**	1	0.5
**FBXW7**	1	0.5
**FGF14**	1	0.5
**FGF6**	1	0.5
**FLT3**	1	0.5
**HGF**	1	0.5
**IKBKE**	1	0.5
**JAK2**	1	0.5
**KDM6A**	1	0.5
**KRAS**	1	0.5
**MAP2K2**	1	0.5
**MDM2**	1	0.5
**MDM4**	1	0.5
**MEN1**	1	0.5
**NF2**	1	0.5
**NOTCH1**	1	0.5
**NTRK3**	1	0.5
**PALB2**	1	0.5
**PRKDC**	1	0.5
**RPTOR**	1	0.5
**SOX10**	1	0.5
**STK11**	1	0.5
**TET2**	1	0.5
**ZNF217**	1	0.5
**ZNF703**	1	0.5

Supplemental Table 3: Frequency of Mutated Genes by Site

Gene	Site	Site Not Previously Identified in Breast Tumor	Frequency (no.)	Percent (%)
**TP53**	R248Q		3	3.0
**TP53**	C238W	+	1	1.0
**TP53**	C242fs*5		1	1.0
**TP53**	F109fs*39	+	1	1.0
**TP53**	F134S	+	1	1.0
**TP53**	F270V	+	1	1.0
**TP53**	G2455		1	1.0
**TP53**	G302fs*42	+	1	1.0
**TP53**	H179R		1	1.0
**TP53**	I195fs*52		1	1.0
**TP53**	I195T		1	1.0
**TP53**	K132R		1	1.0
**TP53**	K320*	+	1	1.0
**TP53**	L114fs*10		1	1.0
**TP53**	M133K		1	1.0
**TP53**	N131del	+	1	1.0
**TP53**	P151A		1	1.0
**TP53**	R213*		1	1.0
**TP53**	R273C	+	1	1.0
**TP53**	R273H		1	1.0
**TP53**	R342P		1	1.0
**TP53**	S241fs*5	+	1	1.0
**TP53**	S303fs*42		1	1.0
**TP53**	V10I	+	1	1.0
**TP53**	W53*	+	1	1.0
**TP53**	Y163*	+	1	1.0
**PIK3CA**	H1047R		10	10.1
**PIK3CA**	E545K		5	5.1
**PIK3CA**	H1047L		3	3.0
**PIK3CA**	E729K		1	1.0
**PIK3CA**	G1049R		1	1.0
**PIK3CA**	N345K		1	1.0
**PIK3CA**	Q546R		1	1.0
**GATA3**	*445fs*2+		1	1.0
**GATA3**	A333fs*20		1	1.0
**GATA3**	D336fs*17		1	1.0
**GATA3**	P436fs*11+	+	1	1.0
**GATA3**	S438fs*9+	+	1	1.0

**PIK3R1**	G376R	+	2	2.0
**PIK3R1**	N453_T454insNN		1	1.0
**PIK3R1**	V172fs*7	+	1	1.0
**PIK3R1**	Y580fs*19	+	1	1.0
**BRCA2**	A938fs*21	+	1	1.0
**BRCA2**	K3326*		1	1.0
**BRCA2**	N43fs*1	+	1	1.0
**BRCA2**	V1283fs*2	+	1	1.0
**CDH1**	Q23*		1	1.0
**CDH1**	T823fs*23	+	1	1.0
**CDH1**	Q511fs*10	+	1	1.0
**HER2(ERBB2)**	V777L		2	2.0
**HER2(ERBB2)**	S1050*	+	1	1.0
**NF1**	1679fs*21	+	1	1.0
**NF1**	R304*	+	1	1.0
**NF1**	R1276Q	+	1	1.0
**ESR1**	L536H	+	1	1.0
**ESR1**	Y537S	+	1	1.0
**HRAS**	G12S	+	1	1.0
**HRAS**	G12D		1	1.0
**AKT1**	E17K		1	1.0
**ARID1A**	Q708*	+	1	1.0
**ATM**	K1698R	+	1	1.0
**BRCA1**	Q1447*	+	1	1.0
**CDKN2A**	Y44fs*1	+	1	1.0
**CREBBP**	P858S	+	1	1.0
**DAXX**	S97fs*46	+	1	1.0
**EGFR**	P848L	+	1	1.0
**EPHB1**	A922T	+	1	1.0
**FBXW7**	D112E	+	1	1.0
**FLT3**	L260*	+	1	1.0
**KDM6A**	Q692fs*37	+	1	1.0
**KRAS**	G12S	+	1	1.0
**MEN1**	I85fs*33		1	1.0
**NF2**	K159fs*16	+	1	1.0
**NTRK3**	E412K	+	1	1.0
**PALB2**	S766*	+	1	1.0
**PTEN**	K327fs*16	+	1	1.0
**RET**	C634R	+	1	1.0
**SMAD4**	E330K	+	1	1.0
**SOX10**	S431L	+	1	1.0
**TET2**	S714*	+	1	1.0
